# Novel harmine derivatives for tumor targeted therapy

**DOI:** 10.18632/oncotarget.3276

**Published:** 2015-04-22

**Authors:** Siwen Li, Aqin Wang, Fan Gu, Zhaohui Wang, Caiping Tian, Zhiyu Qian, Liping Tang, Yueqing Gu

**Affiliations:** ^1^ Department of Biomedical Engineering, State Key Laboratory of Natural Medicines, School of Life Science and Technology, China Pharmaceutical University, Arlington, TX, USA; ^2^ Department of Biomedical Engineering, School of Automation, Nanjing University of Aeronautics and Astronautics, Nanjing, China; ^3^ Department of Bioengineering, University of Texas at Arlington, Arlington, TX, USA

**Keywords:** harmine, structural modification, 2DG, met, tumor targeting therapy

## Abstract

Harmine is a beta-carboline alkaloid found in medicinal plant PeganumHarmala, which has served as a folk anticancer medicine. However, clinical applications of harmine were limited by its low pharmacological effects and noticeable neurotoxicity. In this study, we modified harmine to increase the therapeutic efficacy and to decrease the systemic toxicity. Specifically, two tumor targeting harmine derivatives 2DG-Har-01 and MET-Har-02 were synthesized by modifying substituent in position-2, -7 and -9 of harmine ring with two different targeting group2-amino-2-deoxy-D-glucose (2DG) and Methionine (Met), respectively. Their therapeutic efficacy and toxicity were investigated both *in vitro* and *in vivo*. Results suggested that the two newharmine derivatives displayed much higher therapeutic effects than non-modified harmine. In particular, MET-Har-02 was more potent than 2DG-Har-01 with promising potential for targeted cancer therapy.

## INTRODUCTION

There is an increasing interest in discovering novel antitumor agents from natural resources. Harmine (7-methoxy-1-methyl-9H-pyrido[3, 4-b]indole), a naturally occurring β-carboline, was previously isolated from a medicinal herbal PeganumHarmala traditionally used in the Middle East and North Africa [1, 2]. Studies in the past decade revealed that harmine possesses potent anti-proliferative and cytotoxic properties. However, harmine's severe neurotoxic effects in animal models impeded its development toward the clinical wide application [3–5].

As a small molecule, harmine may easily penetrate into cells by diffusion. However, harmine shows no tumor specificity, which may be responsible for its weak anti-cancer effects and serious systemic side effects [6–7]. To overcome such drawbacks, intensive research efforts have been placed on the design and synthesis of novel β-carboline derivatives with limited success [8–17]. This work summarizes our efforts on the production of harmine derivatives via targeted modifications to improve its cancer targeting effects.

The tumor targeting natural product derivatives have played an important role for the enhanced tumor therapy [18–22]. Our previous studies demonstrated that 2-amino-2-deoxy-D-glucose (2DG) and methionine (Met) exhibited wide-spectrum tumor targeting ability due to the high metabolic process in various tumor cells [23–24]. It has been commonly acknowledged that the introduction of glucose leads to increased efficiency in tumor uptake [25]. Indeed, ^18^FDG as tumor targeting agent has been widely applied in clinicaldiagnosis [26–29]. It is well established that 2-amino-2-deoxy-D-glucose (2DG), a glucose analog, can be recognized and transported into the cells by GLUT1 on the cell membrane [30]. The absence of hydroxyl group in position 2 of 2DG hampers the isomerization in the enzyme-catalytic metabolic pathway and results in the retention ofphosphorylated molecule within the cell.

Methionine (Met), a sulfur containing essential amino acid indispensable for the growth and development of mammals, has been shown to play many critical roles in mammalian metabolism [31]. Methionine is transported by LAT1 and LAT2 receptors which are highly expressed in many malignant cell membranes [32]. Because of the metabolic defect in methionine production, exogenous methionine is essential to the growth of many malignant cell lines whereas the growth of non-cancerous cells is generally methionine independent. Therefore, 2DG and Met were used in this study as targeting groups to produce harmine derivatives with improved tumor selectivity.

Studies have proved that a high cytotoxic potency is associated with compounds placing hydrophobic bulky groups in position-2, -7 and -9 of harmine ring. The modifications of -2 and -9 site of harmine could enhance the antitumor activity. Thus, the methoxy-substituent at position-7 of beta-carboline might contribute to the significant neurotoxic side effect. On the other hand, the prolonged substituents at position-7 eliminated neurotoxic effects completely [9–10].

In this study, we designed and synthesized two novel targeting antitumor agents bearing 2DG and Met in position-7, phenpropyl in position-9 and benzyl in position-2 of harmine ring. The antitumor activity and neurotoxicity of these novel compounds were assessed. Subsequently, the cell targeting mechanism of the new harmine derivatives was investigated.

## RESULTS

### Synthesis and characterization of 2DG-Har-01 and MET-Har-02

**Figure 1:**
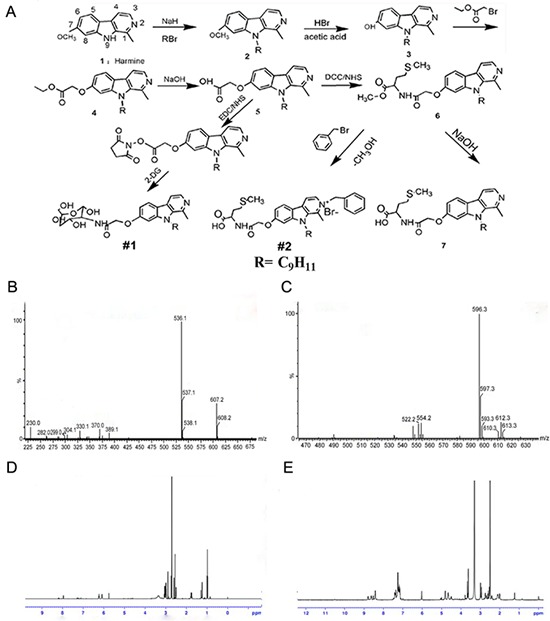
**A.** synthetic scheme of harmine derivatives 2DG-Har-01 and MET-Har-02. **B.** and **C.** Mass spectrums of 2DG-Har-01 (B) and MET-Har-02 (C) **D.** and **E.** ^1^H-NMR spectrums of 2DG-Har-01 (D) and MET-Har-02 (E).

2DG-Har-01 and MET-Har-02 were synthesized by following the series of steps as depicted (Fig. 1A). The N^9^-alkylated harmine derivative 2 was prepared according to the synthetic protocol described before [33]. The preparation of compounds 3 followed a common synthetic scheme [34], characterized by demethylation of compounds 2 using acetic acid and hydrobomic acid as reaction solvent. To modify the molecules with 2DG and Met, a key intermediate, the alky halide, was designed. Compound 4 was obtained by O-alkylation of compound 3 in the presence of cesium carbonate with the ethyl bromoacetate. Then, hydrolyzation of compound 4 lead to the production of –COOH-functionalized compound 5. Finally, 2DG-Har-01 and compound 6 were synthesized with the conjunction of 2DG and Met, respectively. MET-Har-02 is the N^2^-benzylated product of compound 6. The successful synthesis of 2DG-Har-01 and MET-Har-02 were evidenced by MS (Fig.1B, 1C) and ^1^H NMR (Fig. 1D, 1E). The molecular weights of 2DG-Har-01 and MET-Har-02 are 536 and 596, respectively (Fig. 1B, 1C).

### Proliferative inhibition assay

**Figure 2:**
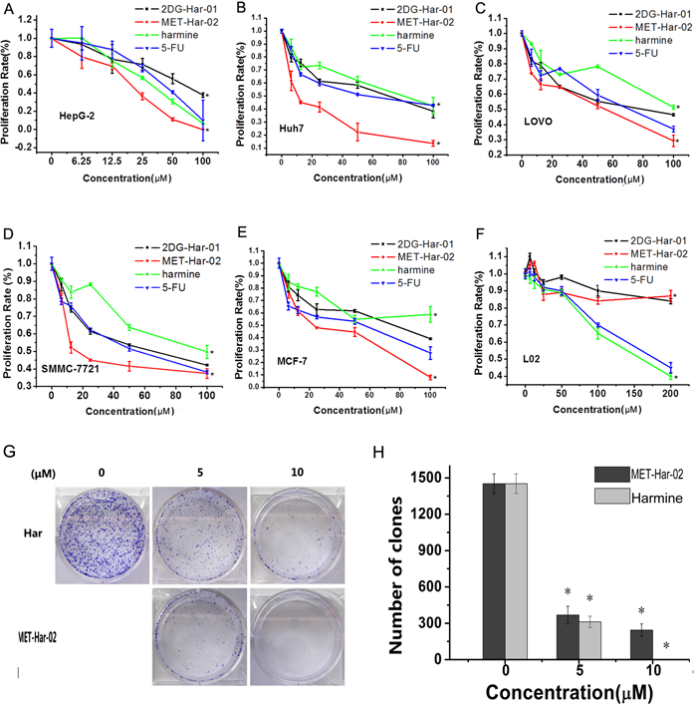
**A–F.** *In vitro* antitumor efficacy and cytotoxicity of 2DG-Har-01 and MET-Har-02. Tumor cell proliferation rate of HepG-2 cells A. Huh7 cells B. LOVO cells C. SMMC-7721 cells D. MCF-7 cells E. and normal liver cell L02 F. incubated with either 2DG-Har-01, MET-Har-02, harmine or 5-FU. **G.** and **H.** Clone formation assay was performed on HepG2 cells. The cells incubated with either harmine (Har) or MET-Har-02 with different concentration (0, 5 10 μM). Data are given as mean ± SD (*n* = 5). *P < 0.05.

We investigated the anticancer effects of 2DG-Har-01, MET-Har-02 on five human cancer cell lines (SMMC-7721, HuH7, HepG2, LOVO and MCF-7). The original harmine and common recognized anticancer drug 5-fu were used as control. As shown in Fig. 2A-2E, the modified harmine derivatives, 2DG-Har-01 and MET-Har-02, showed dose-dependent anti-tumor activity in different concentrations. 2DG-Har-01 showed improved antitumor activities than harmine controls on LOVO and SMMC-7721 cells. On the other hand, MET-Har-02 displayed stronger antitumor activity than harmine controls and 5-fu on all cell lines, especially hepatocellular carcinoma cells (SMMC-7721, HuH7).

Most importantly, unlike harmine control and 5-fu, the targeting compounds 2DG-Har-01 and MET-Har-02 showed slight cytotoxicity on the normal human liver cell L-02 even at the highest concentration (200 μM) (Fig. 2F). These highly selective toxicity results support that 2DG-Har-01 and MET-Har-02 might be uptaken mostly by cancer cells rather than normal cells. The effect of MET-Har-02 and harmine on cancer cells was further investigated using clone formation assay on HepG2 cells. After treated with indicated concentration of MET-Har-02 and harmine for 7 days, the number of clones formed was significantly decreased compared with the control group, (Fig. 2G, 2H). At 5 μM, both MET-Har-02 and harmine were found to inhibit HepG2 cells’ colony formation while MET-Har-02 showed superior suppression effect than harmine. No colony was found when the concentration of MET-Har-02 was raised to 10 μM.

### The apoptosis assay of harmine and MET-Har-02

**Figure 3:**
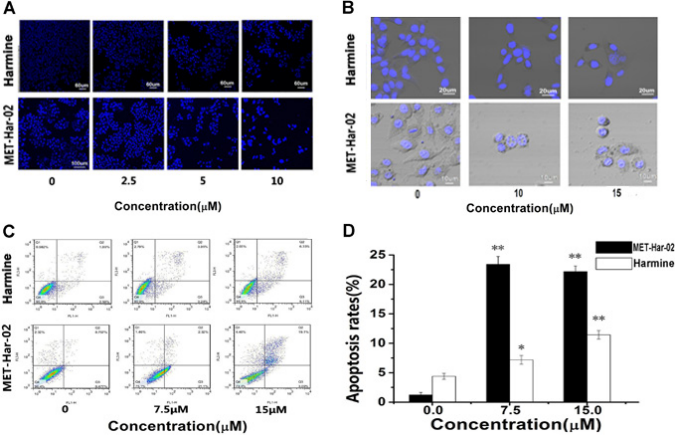
The apoptosis in HepG2 cancer cells after incubated with MET-Har-02 and harmine **A and B.** Morphological changes in apoptotic cells were examined by LSFM microscopy after hoechst staining (original magnification, × 100). **C.** Flow cytometry-based annexin V-FITC/PI labeling of apoptotic cells. **D.** The histogram represents apoptosis rates. Data are given as mean ± SD (*n* = 10). **P < 0.01.

Further studies were carried out to seek the potential processes governing the anti-tumor effect of MET-Har-02 and harmine. Since many anticancer drugs cause the death of tumor cells through the induction of apoptosis, we thus assume that MET-Har-02 promotes cancer cell apoptosis. This hypothesis was tested using HepG2 cell line. As expected, the treatment of MET-Har-02 and harmine both decreased the survival of HepG2 Cells (stained with hoechst) and the cell survival rates were decreased with increasing the compounds concentrations (from 0 to 10 μM) (Fig. 3A). It also suggested that the antitumor activity of MET-Har-02 is better than the hamrine's (the amounts of the cells in the same concentration).

Morphological analysis was performed after MET-Har-02 and harmine treatment for 48 hours. HepG2 Cells were stained with Hoechst33342 for 20 mins and observed through the confocal laser scanning microscope. As shown in (Fig. 3B). The density of cells was decreased with the increasing concentration of the compounds. Cells in the control group fully stretched and showed normal volume. Morphological changes such as cell shrinkage, rounding, nuclear condensation and margination were observed when the cells were treated with MET-Har-02 at 10 μM and 15 μM, but not very obvious in the harmine group.

Annexin V-FITC/PI double labeling method was used to detect apoptosis of HepG2 cells after harmine and MET-Har-02 treatments. Annexin V specifically recognized the cells entering apoptosis that expressed phosphatidyl serine on the outer layer of the cell membrane. Necrotic cells can be stained only with PI, early apoptotic cells with annexin V and late apoptotic cells with both annexin V and PI. Double staining by annexin V and PI allows the discrimination of apoptotic cells from necrotic cells. After treatment of MET-Har-02 in concentration of 7.5 μM for 24 h, the amount of cells in the right bottom quadrant (cells in early apoptosis) increased by 21% (Fig. 3C and 3D). When the concentration of MET-Har-02 was increased to 15 μM, almost the entire early apoptotic cells were transformed into late apoptosis (Fig. 3C and 3D). Apoptosis was also observed in cells treated with harmine. Even at the presence of 15 μM harmine, only 7% cell apoptosis was observed (Fig. 3D). The early apoptosis to late apoptosis was estimated to be 1:1 ratio.

### *In vivo* antitumor activity of 2DG-Har-01 and MET-Har-02

**Figure 4:**
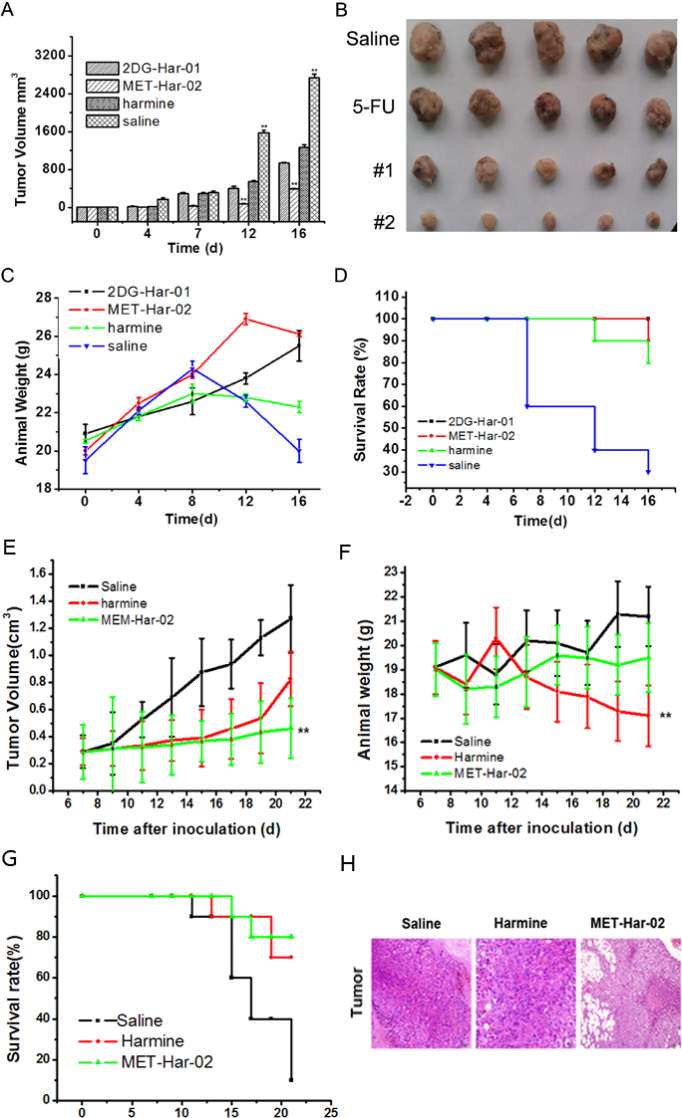
*In vivo* antitumor efficacy of 2DG-Har-01 and MET-Har-02 on S180 tumor-bearing mice and MCF-7 tumor-bearing nude mice **A.** tumor volume of mice-bearing S180 tumors under different treatments (2DG-Har-01, MET-Har-02, harmine and saline, *n* = 10/group). **B.** tumor picture of mice-bearing S180 tumors in different groups (2DG-Har-01, MET-Har-02, harmine and saline) on the 16th day after injection. **C.** body weights of mice bearing S180 tumors in different groups. **D.** the 16-day survival rates of mice after administration of 2DG-Har-01, MET-Har-02, harmine and saline. **E.** tumor volume of nude mice-bearing MCF-7 tumors under different treatments (MET-Har-02, harmine and saline, *n* = 10/group). **F.** body weights of mice bearing MCF-7 tumors in different groups. **G.** the 21-day survival rates of mice after administration of MET-Har-02, harmine and saline. **H.** the histology examination of tumor on different group.

The *in vivo* anti-tumor efficiency of 2DG-Har-01 and MET-Har-02 were evaluated in S180 tumor-bearing mice following the procedures described in section method. As shown in Fig. 4A, the tumor growth was significantly reduced in the mice groups treated with 2DG-Har-01, MET-Har-02 and Harmine compared with saline-injection control group. Among all treatments, MET-Har-02 showed highest tumor inhibition ratio (67.86%) compared to 2DG-Har-01 (42.84%) and Harmine (30.93%) treatment. The tumors isolated from the subject mice after 16 days treatment confirmed the *in vivo* tumor measurements (Fig. 4B). Body weight and survive rate usually reflect the health condition of the treated mice. The body weight of mice in the control group (saline-treated) began to decrease from the 8^th^ dayafter injection (Fig. 4C), which indicated the living quality of the mice was compromised by the tumor burden. For the 2DG-Har-01 and MET-Har-02 treated groups, the body weight gradually increased during the treatment period, implying that the systemic toxicity was minimal in these mice. A slightly decrease of body weight in the harmine group was observed at the end of the treatment period. During the *in vivo* studies of 16 days (Fig. 4D), no mice died with the 2DG-Har-01 treatment. For the same duration, the survival rate of MET-Har-02 group was 90% and harmine group was 80% while only 30% of animals survived with saline injection. These results support that the newly synthesized targeting reagents 2DG-Har-01 and MET-Har-02 treatment may prolong the life expectance of S180 tumor-bearing mice.

Similarly the antitumor efficacy of MET-Har-02 was also investigated on the MCF-7 tumor bearing nude mice. As shown in Fig. 4E, the tumor volume indicated that MET-Har-02 exhibited higher tumor inhibition ratio than harmine, with the inhibitionratios of 63.5% (MET-Har-02) and 35% (harmine). The body weights of subjected nude mice were plotted in Fig. 4F. Compared to the saline group, the MET-Har-02 group didn’t show obvious reduction of weight during the 21 days. The 21-day survival rates of micebearing MCF-7 tumors in the MET-Har-02 and harmine groups were 80% and 70%, respectively, whereas in the control group only one nude mouse was still alive on the 21^th^ day (Fig. 4G). We also performed the pathological examination on the tumor tissues (Fig. 4H). The result showed pronounced pathological changes in the MET-Har-02 group. The most amounts of the tumor cells were dead in the central of the issue and the volume of the surrounding cells decreased obviously, which was similar to the results of antitumor efficacy in the study above.

### Neurotoxic and acute toxicities assay on normal mice

Neurotoxic of 2DG-Har-01, MET-Har-02 and harmine were evaluated both *in vivo* and *in vitro*.

**Figure 5:**
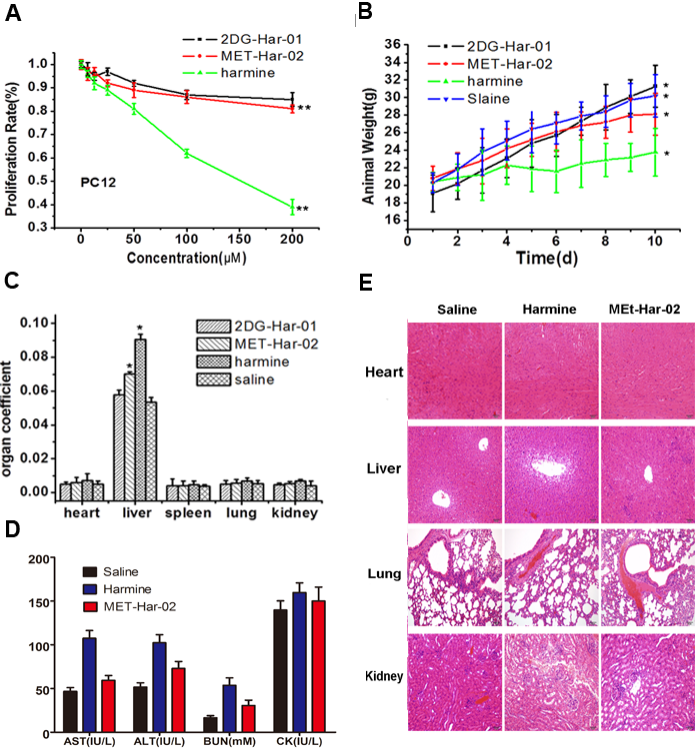
The neurotoxic and acute toxicities of 2DG-Har-01 and MET-Har-02 on healthy mice **A.** the proliferation rate of the mice neural cells PC12 incubated with 2DG-Har-01, MET-Har-02 and harmine. Data are given as mean ± SD (*n* = 10). **P < 0.01. **B.** animal weights of normal mice in different groups (2DG-Har-01, MET-Har-02, harmine and saline). **C.** organ coefficient (organ weight/animal weight) of normal mice in different groups (2DG-Har-01, MET-Har-02, harmine and saline) on the 10th day. **D.** the blood serum index (AST, ALT, BUN, CK) of the mice after the acute toxicities test. **E.** The histology examination on main organ of different mice group (MET-Har-02, harmine and saline treated).

As shown in Table 1, the healthy mice treated with 2DG-Har-01 and MET-Har-02 (10, 50, 100, 200 and 500 mg/kg, i.p.) did not exhibit signs of neurotoxicity, such as tremor, tetanus, twitch, jumping, supination and death during the 10-day observation. As expected, the mice receiving 100 mg/kg harmine exhibited neurotoxic behaviors immediately (Table 1). Moreover, the nerve cell toxicity of these compounds was assessed using PC12 rat neural cells and MTT assay (Fig. 5A). The study showed that the cell survival rate of harmine treated group was 39%, while 2DG-Har-01 and MET-Har-02 treated groups were more than 80% at the drug concentration of 200 μM. These observations suggested that the modified harmine derivatives 2DG-Har-01 and MET-Har-02 have reduced neural toxicity.

**Table 1:** Neurotoxic behaviors of the mice treated with 2DG-Har-01, MET-Har-02 and harmine

	Tremor	Tetanus	Twitch	Jumping	Supination
2DG-Har-01	-	-	-	-	-
MET-Har-02	-	-	-	-	-
Harmine	√	√	√	√	√

Further, the acute toxicities were investigated. Compared with the saline control group, the animal body weight after the treatments of 2DG-Har-01 and MET-Har-02 even at the high concentration of 500 mg/kg displayed a regular increasing trend, without obvious toxicity (Fig. 5B). In contrast, harmine treated mice group exhibited the inhibited growth (Fig. 5B). Further study was carried out to determine the organ coefficient (ratio of organ weight/body weight) of the major organs (heart, liver, spleen, lung and kidney) in animals treated with 500 mg/Kg 2DG-Har-01, MET-Har-02, in the 10^th^ day after injection (Fig. 5C). As expected and in consistent with the animal weight results, the administration of harmine significantly increased liver weight indicating liver toxicity (Fig. 5C). On the other hand, two modified harmine derivatives did not render any significant changes in the organs compared with the control group, except a slight swelling of liver in MET-Har-02 treated animals. To further study the acute toxicity, the serum levels and pathological assay for the main organs were investigated at 10 days after injection of different compounds. As shown in Fig. 5D, the serum levels of AST, ALT, CK and BUN in the harmine treated mice group demonstrated significant elevation (*p* < 0.01), while only slight increasing were observed in the MET-Har-02 treated mice group for AST, ALT, BUN. The levels of CK induced by MET-Har-02 were almost the same with the control group, indicating low toxicity of the modified compound MET-Har-02. To confirm the toxicity, we also did the histological examination for the main organs (heart, liver, lung and kidney) from the above three groups (10/group) (Fig. 5E). The heart and lung showed no obvious pathological changes in mice of all the three groups. In the liver examination, no pathological damage was observed in the saline and MET-Har-02 group, but a little hepatic edema was found in the harmine group. The observation of kidney revealed a little pathological damage in both harmine and MET-Har-02 groups. However, the damage in harmine group was more serious than that in the MET-Har-02 group.

## DISCUSSION

In this study, we presented the derivative synthesis of harmine, a beta-carboline alkaloid, with both molecular modification and targeting agent conjunction [10]. The purpose of the molecular alteration was to acquire new compounds of highly potent antitumor activity and low neural and systemic toxicity. Previous studies have concluded that a higher antitumor efficacy potency of harmine is associated with the compounds bearing hydrophobic bulky groups in position-7 and -9. However, the methoxy substituent at position-7 of beta-carboline might be responsible for their severe neurotoxic effect. To eliminate the neural toxic effects, harmine derivatives were synthesized with prolonged substituents at position-7. The 2DG and MET as the tumor targeting agent were documented in our previous studies. Indeed, the conjugation of 2DG at position-7 using a reaction linkage with methyl bromoacetate significantly increase the tumor cell uptake ability and decrease the neurotoxicity effect. And furthermore we added a hydrocinnamyl at position-9 to enhance the cytotoxicity of the compounds [2, 8]. As anticipated, our results showed that 2DG-Har-01 had better antitumor activity than harmine. To further improve the anti-tumor property, MET-Har-02 bearing N_2_-benzylated modification in position 2 was produced. Benzyl in position-2 of harmine was reported to have significant contribution to the anti-proliferation effect of harmine derivatives [3, 8, 10, 20]. It should be noted that, possibly due to the steric hindrance of 2DG, benzyl cannot be modified on the position-2 of 2DG-Har-01 (the stereospecific blockade and demonstrated by our study). Both our *in vitro* and *in vivo* results support that both derivatives, MET-Har-02 and 2DG-Har-01, have superior antitumor property and cell/animal safety than harmine.

These all *in vitro* and *in vivo* experiment results have manifested that structural modification in position 2, 7, 9 of harmine could improve the antitumor activity and decrease the side effects of the compounds. The reason of the ideal results is that the targeting agent modifications reduce the uptake of other organs and the molecular modifications enhance the antitumor activity to the cancer cells.

Subsequent work was emphasized on identifying the antitumor mechanism of MET-Har-02. Since harmine and many other beta-carboline agents have been shown to kill tumor cells by launching apoptosis [21], we have hypothesized that MET-Har-02 trigger cancer cells undergone apoptosis. This hypothesis is supported by several observations. Firstly, Annexin V-FITC/PI double labeling assay showed that different concentration of MET-Har-02 induced apoptosis on different phases. These results were also confirmed by Hoechst staining. Second, while non-treated cells fully stretched and showed normal volume, treated ones showed typical apoptotic cell morphology with shrunk, turned round, presented condensed nuclear and margination. These finding supports our assumption that the anti-proliferative effects of MET-Har-02 may be attributable to its induction of apoptotic cell death.

Apoptosis may be mediated by different pathways. Briefly, caspase, a family of cysteine proteases, is an integral part of the apoptotic pathway. Moreover, mitochondria mediated apoptosis is precisely regulate by Bcl-2 family proteins. Furthermore, the Bcl-2/Bax ratio is known as the ‘molecule trigger’ of apoptosis [35]. Interestingly, MET-Har-02 has insignificant influence on the ratio of Bcl-2/Bax. These results indicate that MET-Har-02 may have no effect on the Bcl family proteins in HepG2 cell at the test condition (Supplementary materials 1)

It is well known that cell apoptosis and the cell cycle are tightly linked. According to former researches, several beta-carboline compounds, including harmine, change the cell cycle thorough specific inhibition of CDKs which are crucial molecules in the regulation of different phases of the cell cycle [36]. Our data showed that MET-Har-02 caused cell cycle arrest in the G1 phase of HepG2 cell, which was distinct from the known antitumor effect of harmine. In addition, wound scratch results have shown that the MET-Har-02 suppresses tumor metastasis even at a low concentration of 5 μM (Supplementary materials 2).

## MATERIALS AND METHODS

### Reagents

Harmine (MW 212.24, ≥ 98% purity) was purchased from Xi’AnFeida Bio-Tech CO. LTD (Xi’An China). All solvents and reagents used in this study were analytical reagent grade, and obtained from Aladdin (Shanghai, China). Silica gel GF_254_ was used in analytical thin-layer chromatography (TLC) and silica gel was used in column chromatography. D-(+)-Glucosamine hydrochloride (MW 164.16) and Methionine (MW 149.21), RPMI-1640, dimethylsulfoxide (DMSO), hoechst 33342 and 3-(4, 5-dimethylthiazol-2-yl) -2, 5-diphenyl tetrazolium bromide (MTT) fetal bovine serum (FBS), streptomycin, penicillin, and trypsin-EDTA were purchased from commercial sources. Antibodies specific for caspase-8 were purchased from Cell Signaling Technology (city, country). Antibodies specific for caspase-9, Bcl-2, Bcl-xL, and GAPDH were purchased from Epitomics (Zhejiang, Nanjing).

### Cell lines and cell culture

Human hepatoma cell lines SMMC-7721, HuH7, HepG2, human pancreatic cancer cell line PC-3, human lung cancer cell A549, human breast cancer MCF-7 and immortal hepatic cell lines L02 cell line, human colorectal LOVO cell line were purchased from Cell Bank of Shanghai Institute of Biochemistry & Cell Biology, Chinese Academy of Sciences (Shanghai, China). All cell lines were routinely cultured at 37°C, 5% CO2 in RPMI 1640 or DMEM with 10% fetal bovine serum (FBS), 5% penicillin/streptomycin.

### Animal subjects

All normal (Kunming) mice were purchased from Charles River Laboratories (Shanghai, China). S180 sarcoma cell lines were provided by Shanghai Institute of Pharmaceutical Industry. All animal experiments were carried out in compliance with the Animal Management Rules of the Ministry of Health of the People's Republic of China (document NO. 55, 2001) and the guidelines for the Care and Use of Laboratory Animals of China Pharmaceutical University.

### Methods

Synthetic routine of 2DG-Har-01 and MET-Har-02 were described as scheme 1 (Fig. 1A). All synthetic steps were divided into three parts. Part I: Modification ofharmine in positon-7 and- 9, gave harmine derivatives (2), (3), (4) and non-targeted harmine derivative (5). Part II: Targeting modification of harmine derivatives with 2DG and Met provided 2DG-Har-01 and compound (6), respectively. Part III MET-Har-02 was finally prepared through Modification of compound (6) in position-2.

### Synthesis of 7-methoxy - 9-(3-phenylpropyl)- 1-methyl-β-carboline (compound 2)

A mixture of 7-methoxy-1-methyl-β-carboline (compound **1**:harmine) (0.530 g, 2.5 mmol), anhydrous DMF(15 mL) and NaH (0.090 g, 3.75 mmol) was stirred about 5 minutes at room temperature (RT) until it was clear, and then 3-phenylpropyl bromide (0.995 g, 5 mmol) was added. The mixture was stirred continually about 3–4 h at RT. After completion of the reaction as indicated by TLC (petroleum ether/acetone1:1), the solution was poured into cold water (30 mL), stirred for 20 minutes, and then the yielded precipitate was filtered and washed with considerable water. The precipitate was dissolved in ethanol and adjusted to pH 3–4 by HCl, and then the ethanol was removed on the rotary evaporator. The white solid was recrystallized from acetone, and hydrochloride of compound 2 was obtained.

Yield = 100%, R_f_ = 0.55 (petroleum ether/acetone1:1) ESI-MS m/e (M + 1) 331.2.IR (KBr, cm^−1^) 2059, 1619, 1544, 1473, 1441, 1407, 1345, 1250, 1207, 1140, 1034, 813, 743

^1^H-NMR (500 MHz, CDCl3) δ8.23–8.24 (1 H, d, J = 5.1 Hz); 7.81–7.82 (1 H, d, J = 5.13 Hz); 8.10–8.08 (1 H, d, J = 8.6 Hz); 7.11–7.12 (1 H, m); 6.86–6.88 (1 H, m); 7.11–7.30 (5 H, m); 3.90 (3 H, s); 3.15 (3 H, s); 2.01–2.03 (2 H, m); 4.52–4.55(2 H, m); 2.70–2.73(2 H, m)^13^ C-NMR (500 MHz, CDCl3) δ163.7, 146.2, 139.7, 135.9, 133.7, 133.6, 128.8, 128.7, 128.3, 126.6, 123.8, 113.4, 112.8, 92.7, 76.9, 55.8, 44.2, 32.5, 31.5, 17.2.

### Synthesis of 7-hydroxy - 9-(3-phenylpropyl)- 1-methyl-β-carboline (compound 3)

Hydrobromic acid 40% aqueous (20 mL) was added to the solution of hydrochloride of compound **2** (0.825 g, 2.5 mmol) in glacial acetic acid (20 mL). The solution was heated to reflux for 12 h. After completion of the reaction as indicated by TLC, the mixture was cooled and poured onto ice. The aqueous mixture, made basically with sodium hydroxide and sodium bicarbonate, yielded a precipitate that was filtered and dried. The solid was crystallized from anhydrous ethanol. White crystals were obtained.

Yield = 95%, R_f_ = 0.58 (petroleum ether/acetone1:1). ESI-MS m/e (M + 1) 317.4. IR (KBr, cm^−1^) 3471–2200, 1640, 1565, 1493, 1411, 1338, 1247, 1194, 1164, 1117, 1021, 807, 747 1H-NMR (500 MHz, CDCl3) δ9.72 (1 H, s, OH); 8.23–8.24 (1 H, d, J = 5.1Hz); 7.81–7.82 (1 H, d, J = 5.13 Hz); 8.10–8.08 (1 H, d, J = 8.6 Hz); 7.11–7.12 (1 H, m); 6.86–6.88 (1 H, m); 7.11–7.30 (5 H, m); 3.15 (3 H, s); 2.01–2.03 (2 H, m); 4.52–4.55 (2 H, m); 2.70–2.73 (2 H, m).^13^ C-NMR (500 MHz, CDCl3) δ157.4, 137.7, 129.8, 128.6, 128.4, 126.3, 122.5, 112.2, 109.7, 95.4, 77.2, 76.9, 76.7, 44.2, 33.0, 31.9, 22.5

### Synthesis of 7-(ethoxycarbonylmethoxylene)- 9-(3-phenylpropyl)-1-methyl-β-carboline (compound 4)

Ethyl bromoacetate (0.594 g 3.56 mmol) and cesium carbonate (1.158 g 3.56 mmol) were added to compound **3** (0.749 g 2.37 mmol) dissolved in anhydrous dimethylformamide (DMF) (20 mL). Then, the reaction mixture was stirred at room temperature for 15–24 hours. After completion of the reaction as indicated by TLC, the mixture was cooled and diluted with dichloromethane, washed once with water and three times with brine. The organic layer was dried over Na_2_ SO_4_ and concentrated. The crude product was purified using column chromatography (dichloromethane/ethanol v/v 30:1).

Yield = 75%, R_f_ = 0.5 (petroleum ether/acetone1:1). ESI-MS m/e (M + 1) 403.4. IR (KBr, cm^−1^) 2957, 2921, 1765, 1624, 1575, 1472, 1437, 1386, 1205, 1079, 815, 745

1H-NMR (500 MHz, CDCl3) δ8.23–8.24 (1 H, d, J = 5.1 Hz); 7.81–7.82 (1 H, d, J = 5.13 Hz); 8.10–8.08 (1 H, d, J = 8.6 Hz); 7.11–7.12 (1 H, m); 6.86–6.88 (1 H, m); 7.11–7.30 (5 H, m); 3.15 (3 H, s); 2.01–2.03 (2 H, m); 4.52–4.55 (2 H, m); 2.70–2.73 (2 H, m); 4.71 (2 H, s); 4.30–4.34 (2 H, m); 1.32–1.35 (3 H, m)^13^ C-NMR (500 MHz, CDCl3) δ158.6, 153.1, 149.4, 140.1, 138.8, 128.3, 128.6, 125.8, 121.5, 120.6, 120.2, 107.3, 96.6, 75.6, 71.0, 59.5, 58.3, 33.5, 31.3, 16.4, 13.6.

### Synthesis of 7-(oxyacetoxy)- 9-(3-phenylpropyl)-1-methyl-β-carboline (compound 5)

A mixture of compound **4** (0.716 g 1.78 mmol), NaOH (0.2 g 5 mmol), ethanol (10 mL) and H_2_O (20 mL) was refluxed for 2 h, and the ethanol was removed on the rotary evaporator. The mixture was neutralized (pH 5) with 5 M HCl and cooled. The precipitate was collected, washed well with H_2_O and dried in vacuum, then the yellow solid compound 5 was obtained.

Yield = 99%, ESI-MS m/e (M + 1) 375.4. IR (KBr, cm^−1^) 3445–2851, 1654, 1624, 1576, 1458, 1411, 1312, 1231, 1139, 1055, 825, 760

1H-NMR (500 MHz, CDCl3) δ8.23–8.24 (1 H, d, J = 5.1 Hz);7.81–7.82 (1 H, d, J = 5.13Hz); 8.10–8.08 (1 H, d, J = 8.6 Hz); 7.11–7.12 (1 H, m); 6.86–6.88 (1 H, m); 7.11–7.30 (5 H, m); 3.15 (3 H, s); 2.01–2.03 (2 H, m); 4.52–4.55 (2 H, m); 2.70–2.73 (2 H, m); 4.71 (2 H, s) ^13^C-NMR (500 MHz, CDCl3) δ170.1, 159.1, 142.5, 141.0, 140.4, 137.6, 134.5, 128.2, 125.8, 122.2, 114.4, 112.1, 109.3, 94.4, 65.5, 43.5, 40.0, 39.6, 32.0, 31.5, 22.7.

### Synthesis of N-(9-phenylpropyl-1-methyl-β-carboline 7-oxyacetyl)-(2-amino-2-deoy) - Glucose (2DG-Har-01)

Compound **5** (0.329 g 0.88 mmol) was activated with EDCI (0.253 g 1.32 mmol) and N-hydroxysuccinimide (0.152 g 1.32 mmol) in DMF (10 mL). After the mixture was stirred at room temperature overnight, the solution was added with aqueous solution of D-(+)-glucosamine hydrochloride (0.379 g 1.76 mmol), and the reaction mixture was stirred at room temperature for another 12 hours. After completion of the reaction as indicated by TLC, the DMF was removed on the rotary evaporator. The crude Glucose (2DG-Har-01) was further purified using column chromatography (dichloromethane/ethanol v/v10:1).

Yield = 52%, R_f_ = 0.7 (dichloromethane/ethanol v/v15:7). ESI-MS m/e (M + 1) 536.5. IR (KBr, cm^−1^) 3600–3100, 2955, 2923, 2852, 2115, 1777, 1712, 1643, 1555, 1464, 1377, 1263, 1082, 971, 719

1H-NMR (500MHz, DMSO) δ8.23–8.24 (1 H, d, J = 5.1 Hz); 7.81–7.82 (1 H, d, J = 5.13 Hz); 8.10–8.08 (1 H, d, J = 8.6 Hz); 7.11–7.12 (1 H, m); 6.86–6.88 (1 H, m); 7.11–7.30 (5 H, m); 3.15 (3 H, s); 2.01–2.03 (2 H, m); 4.52–4.55 (2 H, m); 2.70–2.73 (2 H, m); 4.71 (2 H, s); 2.50–3.05 (7 H, m, ringGlu-H)

^13^C-NMR (500 MHz, CDCl3) δ169.6, 161.1, 158.3, 145.4, 140.8, 137.5, 133.5, 132.3, 129.7, 128.2, 125.9, 124.2, 114.1, 113.5, 112.1, 94.5, 65.0, 54.4, 41.9, 36.2, 34.1, 31.4, 28.9, 25.2, 15.5.

### Synthesis of N-(9-phenylpropyl-1-methyl-β-carboline 7-oxyacetyl)- L-Methionine methyl ester (compound 6)

Compound **5** (0.329 g 0.88 mmol) was activated with DCC (0.272 g 1.32 mmol) and N-hydroxysuccinimide (0.152 g 1.32 mmol) in DMF (15 mL). After the mixture was stirred at room temperature overnight, the solution was added with L-Methionine methyl ester hydrochloride (0.289 g 1.76 mmol), and the reaction mixture was stirred at room temperature for another 10 hours. After completion of the reaction as indicated by TLC, the DMF was removed on the rotary evaporator. The compound 6 was purified using column chromatography (dichloromethane/ethanol v/v 25:1).

Yield = 70%, R_f_ = 0.6 (dichloromethane/ethanol v/v 15:1). ESI-MS m/e (M + 1) 520.2. IR (KBr, cm^−1^) 3313, 2957, 2922, 2851, 1743, 1624, 1533, 1455, 1261, 1027, 804, 752

1H-NMR (500 MHz, CDCl3) δ8.36–8.37 (1 H, d, J = 5.0 Hz); 8.09–8.11(1 H, d, J = 10 Hz);7.90–7.91 (1 H, d, J = 5.0 Hz); 7.40–7.43 (2 H, m); 7.35–7.37 (1 H, m); 7.27–7.29 (2 H, d, J = 10 Hz); 7.04–7.06 (1 H, m); 6.80

–6.81 (1 H, d, J = 5.0 Hz); 3.05 (3 H, s); 2.25–2.28 (2 H, m); 4.89–4.93 (2 H, m); 2.84–2.86 (2 H, m); 3.82 (2 H, s); 2.12 (3 H, s); 2.56–2.59 (2 H, m); 4.62–4.69 (2 H, m); 4.52–4.55 (1 H, m); 2.75 (3 H, s)^13^ C-NMR (500 MHz, CDCl3) δ172.7, 171.9, 159.1, 143.8, 140.1, 139.2, 135.2, 135.0, 130.8, 128.8, 128.3, 126.7, 123.3, 115.6, 112.9, 110.3, 94.5, 76.9, 67.7, 52.7, 51.2, 32.8, 31.4, 30.9, 29.3, 20.5, 14.0

### Synthesis of N-(9-phenylpropyl-1-methyl-β-carboline 7-oxyacetyl)- L-Methionine bromate (MET-Har-02)

A mixture of 6 (0.322 g 0.62 mmol) and benzyl bromide (0.632 g 6 mmol) in ethyl acetate (4 mL) was refluxed for 12 h. The reaction mixture was monitored by TLC and then cooled at 0°C. The yellow solid MET-Har-02 was filtered under reduced pressure and washed well with ethyl acetate, and then recrystallized from ethanol, dried in vacuum to give yellow crystals.

Yield = 28%, ESI-MS m/e (M+) 596.2. IR (KBr, cm^−1^) 3405–3028, 2951, 2924, 2855, 2793, 1744, 1622, 1537, 1454, 1343, 1209, 1068, 816, 733, 701. ^1^H-NMR (500 MHz, DMSO) δ6.02–8.80 (15 H, m); 2.99 (3 H, s); 2.10–2.11 (2 H, m); 4.88–4.52 (2 H, m); 2.67–2.68 (2 H, m); 3.78 (2 H, s); 2.59 (3 H, s); 2.73–2.76 (2 H, m); 4.64–4.67 (2 H, m); 5.00–5.03 (1 H, m);^13^C-NMR (500 MHz, DMSO) δ172.6, 171.8, 134.1, 129.3, 128.9, 128.7, 128.3, 128.2, 126.7, 126.7, 77.7, 40.0, 39.8, 39.6, 39.4, 39.3, 39.1, 38.9, 35.1, 34.8, 31.1, 28.9, 28.5, 25.3, 25.1, 21.9

### Characterization of 2DG-Har-01 and MET-Har-02

Q-TOF Micro Mass Spectrometer (Waters), Nuclear Magnetic Resonance Spectrometer (BRUKER) and Fourier Transform Infrared spectroscopy (Waters) were used to validate the successful synthesis of 2DG-Har-01 and MET-Har-02.

### Proliferation inhibition assays

The effect of products 2DG-Har-01 and MET-Har-02 on human cancer cell growth were carried out on HepG2, SMMC-7721, HuH7, LOVO, MCF-7 and L02 using MTT assay according to the following procedures. Cells were plated at a density of 5 × 10^3^ cells/well in 96-well plates and incubated overnight. Cells were then incubated with indicated concentration of 2DG-Har-01 MET-Har-02, or controls (harmine or 5-FU) for 48 h. Five replicates were performed for each concentration. MTT solution (5 mg/mL) was add each well. After incubation for 4 h, the medium was discarded and the formazan crystals was dissolved in 100 μL DMSO. The plates were shaken for 15 min. The optical density (OD) value was measured at 490 nm with a microplate reader. The proliferation rate was calculated by the following equation: proliferation rate (%) = (OD_treated_/OD_control_) × 100%.

### Colony formation assay

HepG-2 cells were trypsinized and seeded in 6-well microplates (500 cells/well) in 2 mL of complete RPMI-1640 and were examined microscopically to confirm that only single cell without clumps had been plated. Then thecellswere treated with theindicated concentrations MET-Har-02 and harmine for 7 days. During colony growth, the culture medium was replaced every 3 days. Finally, the cells were fixed with methanol and stained with crystal violet. Imagesof thecolonies were captured by a digital camera. Colonies were visualized by microscopy with an Olympus microscope (Olympus, BX51, Tokyo, Japan). The colonies with more than 50 cells were counted under light microscope. Colony formation rate was calculated with the equation: colony formation rate = (number of colonies/number of seeded cells). Each test was repeated in triplicate.

### Apoptosis analysis

HepG-2 cells growing on 6-well dishes were treated with chemicals in the presence or absence of harmine and MET-Har-02 for 24 h and total cells were collected. The percentage of apoptotic cells was evaluated by staining 1 × 10^6^ cells with annexin V-FITC (Beyotime) and propidium iodide (Beyotime) in binding buffer for 10 min at room temperature in the dark. The samples were analyzed by flow cytometry (FACScalibur, Becton–Dickinson) within 1 h to determine the percentage of cells displaying annexin V+/propidium iodide-(early apoptosis) or annexin V-/propidium iodide + staining(late apoptosis). Three independent experiments were performed for each assay condition.

### Morphological assay

About 3 × 10^5^ HepG-2 tumor cells were seeded at the confocal petri dish and incubated at 37°C for 24 h and treated with MET-Har-02 for 24 h. Hoechst 33342 (Beyotime) were added in 30 mins advance before laser confocal microscopy observation (LCFM, FluoView^TM^ FV1000, Olympus, Japan)

### *In vivo* antitumor activity analysis by tumor xenograft model

S180 cells (5 × 10^6^) were subcutaneously injected into the Kunming mice (*n* = 10 per group). As the tumors grew up to a diameter of 0.2–0.5 cm, the mice were used for treatment. The active compound 2DG-Har-01 and MET-Har-02 were tested for *in vivo* antitumor activity in the Ehrlich solid carcinoma (S180) assay model, while harmine and normal saline (NS) were used as the positive and negative controls respectively. The effect of 2DG-Har-01 and MET-Har-02 treatments on S180 tumor growth were assessed by measuring tumor volume with a sliding caliper and body weight. We measured tumor volume after 96 h post inoculation. The mice were randomly assigned into four groups, and each group was treated once every other day by injection of 0.2 mL of the solution of 20 mg/kg of 2DG-Har-01, MET-Har-02 and harmine in NS for six times. After treatment, tumor sizes were measured every other day until average tumor volume of control group reached approximate 3000 mm^3^. At this time, all mice were killed and the tumors were weighed. Body weight changes were also monitored every other day. The inhibition ratio (IR) was defined as follows: Inhibition ratio (%) = ((Wc – Wt)/Wc) × 100%. Wc and Wt stand for the average tumor weight for control group and treatment group, respectively.

After the antitumor activity assay performed on the Kunming mice, we also conducted the experiment on the nude mice which were injected with human breast tumor cells MCF-7. Similarly, the tumor bearing nude mice were also divided into 3 groups (n = 10) and treated on days 7, 11, 15 and 19 post –inoculation by the same procedures as Kunming mice group, with the dose of 20 mg/kg harmine and MET-Har-02. The therapeutic efficacies were assessed by measuring tumor volume and body weight every other day till the 21th day and the survival rate were recorded.

### Histology examination

To further investigate the side effects of harmine and MET-Har-02 on various organs of the treated mice, histological analysis of different organs was conducted by the established technique.

### Acute toxicities assay

The acute toxicity of 2DG-Har-01, MET-Har-02 and harmine (as control) to animal subjects were investigated on the normal mice. Briefly, 30 Kunming mice (aged 3–4 weeks, weighed 18–22 g, equal number of male and female subjects) were divided randomly into 4 groups including the control group. Mice in the control group were injected with PBS buffer (0.2 mL). The mice were given an ip injection of 10, 50, 100, 200 and 500 mg/kg of 2DG-Har-01, MET-Har-02 and harmine in 0.2 mL of 0.9% saline. After administration, the mice were observed thoroughly for the onset of any immediate neural toxicities and delayed effects. The mice were monitored continuously for 10 days to observe any abnormal behaviors or death. All animals were sacrificed on the 10^th^ day and checked macroscopically for possible damage to the heart, liver, spleen, lung and kidneys. The weight changes of the tissues and bodies were assessed and compared. Blood was drawn from the eye socket. The serum biochemical parameters including aspartate aminotransferase (AST), alanine aminotransferase (ALT), blood urea nitrogen (BUN) and creatinekinase (CK) indexes of the blood samples were examined.

### Statistical analysis

All data were reported as the mean SD of n independent measurements. Statistical analysis was performed by using Student's *t*-test with statistical significance assigned for *P* value of < 0.05.

## CONCLUSION

Harmine and its reported derivatives present several drawbacks arising from the relative weak antitumor activities and the serious neurotoxicity. To overcome the limitation, we successfully conjugated two novel harmine derivatives with different tumor metabolic specific lignads. The two novel harmine derivatives i.e. 2DG-Har-01 and MET-Har-02 showed higher antitumor efficiency and lower systemic and neural toxicity than un-modified harmine in a broad spectrum of tumor lines. In particular, MET-Har-02 displayed better antitumor activity than that of 2DG-Har-01. Our results demonstrated that MET-Har-02 is a promising candidate for targeted cancer therapy.

## SUPPLEMENTARY FIGURES AND TABLES


