# Supplementary material for: Novel harmine derivatives for tumor targeted therapy
Source: Oncotarget. 2015 Apr 22;6(11):8988–9001. doi: 10.18632/oncotarget.3276 (PMC4496197; doi:10.18632/oncotarget.3276)
Supplement: Supplementary file 1 [file oncotarget-06-8988-s001.pdf]

SUPPLEMENTARY FIGURES

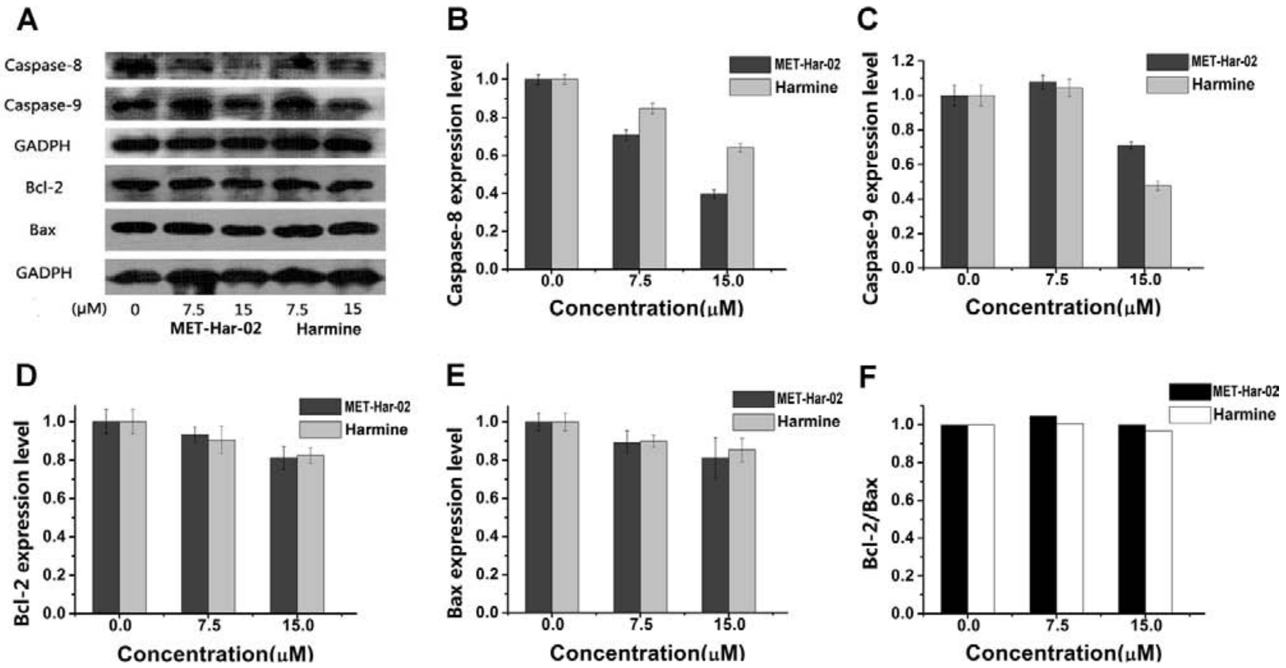

Supplementary Figure S1: A. the western blot analysis of HepG2 cells treated with different dosage MET-Har-02 and harmine. (B-E) the expression level analysis of Caspase-8 **B.** Caspase-9 **C.** Bcl-2 **D.** and Bax **E.** with different dosage MET-Har-02 and harmine. **F.** ratio of Bcl-2 and Bax expression with different dosage MET-Har-02 and harmine. Data are given as mean ± SD (*n* = 3).

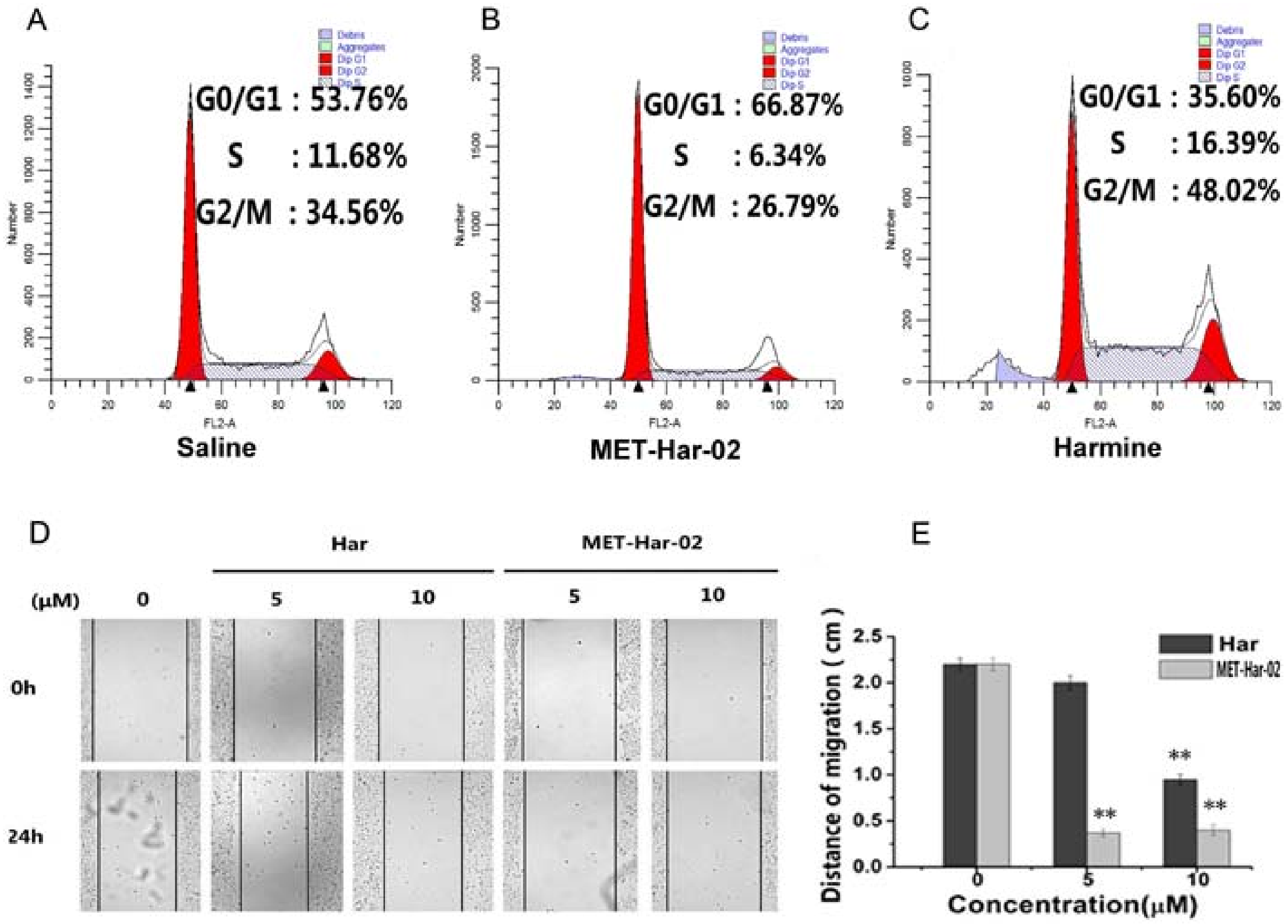

Supplementary Figure S2: (A-C) Cell cycle analysis of HepG2 cells treated with nothing A. MET-Har-02 B. and harmine C. for 24 hours by flow cytometry. D. Wound healing assay of HepG2 cells incubated with harmine and MET-Har-02 for 8 hours in different dosage. **E.** the distance of migration of HepG2 cells in the wound healing assay. Data are given as mean ± SD ($n = 3$). $^{**}P < 0.01$.
